# Mental Health Awareness, Barriers and Support Needs Among Construction Workers: A Service Evaluation

**DOI:** 10.1192/bjo.2026.11541

**Published:** 2026-06-30

**Authors:** Abdul-Adl Bolaji, Chandan Aladakatti, Gabriela FontesDeOliveira, Suleman Patel, Rachel Gregg

**Affiliations:** Midlands Partnership Foundation Trust, Stafford, United Kingdom

## Abstract

**Aims:**

The aim of this service evaluation was to assess baseline mental health awareness, openness to discussing mental health issues, recent experiences and perceived barriers to seeking help among construction workers, and to identify preferred workplace interventions to inform targeted mental health support initiatives.

**Methods:**

An anonymous survey was conducted at a single construction site. Participants completed a questionnaire assessing mental health training exposure, confidence in recognising mental health issues in themselves and in others, openness to talking about mental health problems, awareness of mental health support services, perceived barriers to seeking help, recent mental health experiences and preferred support interventions. Responses were analysed using descriptive statistics.

**Results:**

The survey was completed by 18 construction workers at a single site. Only 56% had received a form of mental-health training. Awareness of support services was variable with 72% knowing about Samaritans, while 56% were aware of the NHS urgent mental-health helpline and the Construction Industry Helpline. Stress at work was reported by 78% of respondents, and approximately 66% experienced low mood, anxiety or sleep disturbance. The most common barriers to seeking help were the belief that others would not care (39%) and stigma or taboo (33%). Preferred interventions included supervisor mental-health training (50%), short educational mental health talks (44%) and mental health champion programmes(39%).

**Conclusion:**

A significant number of construction workers in this survey reported recent experiences with stress, depression and anxiety, alongside gaps in mental health training and awareness of available support. Stigma and belief that others would not care were identified barriers to seeking help, reinforcing the impact that culture and society plays on these perceived barriers. Participants favoured practical, workplace based interventions, particularly supervisor training, brief educational sessions and peer support initiatives. These findings support the implementation of targeted on-site mental health initiatives to improve engagement and accessibility of support within this high-risk occupational group.

